# Not again! Effect of previous test results, age group and reason for testing on (re-)infection with Chlamydia trachomatis in Germany

**DOI:** 10.1186/s12879-018-3323-2

**Published:** 2018-08-25

**Authors:** Alexandra Sarah Lang, Matthias an der Heiden, Klaus Jansen, Andrea Sailer, Viviane Bremer, Sandra Dudareva, Chlamydia trachomatis laboratory sentinel team, Michael Baier, Eberhard Straube, Armin Baillot, Patricia Bartsch, Thomas Miedl, Thomas Brüning, Josef Cremer, Helga Dallügge-Tamm, Arndt Gröning, Stephan Eicke, Dagmar Emrich, Florian Mielack, Martina Woitkowiak, Gundula Fritsche, Rosi Gjavotchanoff, Peter Gohl, Matthias Götzrath, Axel Meye, Ingrid Ehrhard, Beate Köpke, Birgit Henrich, Caroline Kastilan, Susanne Lehmann, Anneliese Märzacker, Stefan Odenbreit, Claudio Perocco, Bernhard Miller, Gerrit Mohrmann, Christian Pache, Roland Pfüller, Carsten Tiemann, Hilmar Wisplinghoff, Thomas Müller, Manuela Wudy, Christian Aepinus

**Affiliations:** 1 Department for Infectious Disease Epidemiology, Robert Koch Institute, Berlin, Germany; 2 Charité Universitätsmedizin Berlin, Berlin, Germany

**Keywords:** Chlamydia trachomatis, Sexually transmitted infections, STI, Reinfection, Screening, Infertility, Sentinel

## Abstract

**Background:**

Infection with *Chlamydia trachomatis* (Ct) is the most commonly reported sexually transmitted infection in Europe. In Germany, Ct screening is offered free of charge to pregnant women since 1995 and to women < 25 years of age since 2008. For symptomatic individuals, testing is covered by statutory health insurance. Study results have shown that repeat Ct infection occurs in 10–20% of previously infected women and men. Our aim was to describe persons tested for Ct and to investigate the determinants of (repeat) Ct infection in women and men in Germany.

**Methods:**

We analysed Ct test results from men and women tested between 2008 and 2014 in laboratories participating in the German *Chlamydia trachomatis Laboratory Sentinel surveillance.* Reinfection was defined as at least 2 positive laboratory tests within more than 30 days. We performed logistic regression stratified by sex and, for women, reason for testing to determine the effect of previous test results and age group on subsequent test results.

**Results:**

In total, 2,574,635 Ct tests could be attributed to 1,815,494 women and 123,033 men. 5% of women and 14% of men tested positive at least once. 15–19- and 20–24-year-old women tested positive at least once respectively in 6.8 and 6.0%, while men respectively in 16.6 and 21.2%. Altogether, 23.1% of tested women and 11.9% of tested men were tested repeatedly between 2008 and 2014. Among those who previously tested positive, reinfection occurred in 2.0% of women and 6.6% of men. Likelihood to be tested Ct positive was higher in women and men with a positive Ct test in the past compared to previously tested Ct negative, odds ratios 4.7 and 2.6 (*p* < 0.01) respectively. Odds ratios ranged by age group and test reason.

**Conclusion:**

A history of Ct infection increased the likelihood of infection with Ct in women and men taking into account the result of the previous test. Health education, safer sex and treatment of partners are necessary for women and men who have tested positive to prevent reinfection and complications and to interrupt the chain of transmission. To identify potential reinfection repeat testing after treatment should be performed.

**Electronic supplementary material:**

The online version of this article (10.1186/s12879-018-3323-2) contains supplementary material, which is available to authorized users.

## Background

Infection with *Chlamydia trachomatis* (Ct) is the most commonly reported sexually transmitted infection (STI) in Europe [1]. According to data of the European Center for Disease prevention and control (ECDC), young people are particularly affected, with two-thirds (67%) of the 384,555 reported cases in 2013 diagnosed amongst 15 to 24-year-olds [2]. This is however strongly influenced by testing practices in reporting countries [2]. In contrast to other STIs, Ct infection is very common in the general population with prevalence ranging from 3.0 to 5.3% among women 18–26-years old and 2.4–7.3% among men 18–26 years old [3]. Population-based estimations for Germany showed prevalence of 4.4% for sexually active 17-year-olds, 4.5% for 18- to 19-year-old women and 4.9% for 25- to 29-year-old men [4]. Overall, women aged 20 to 24 years are the group most frequently diagnosed with Ct [5, 6]. The most consistent risk factors associated with Ct infection in other studies are young age and a high number of sexual partners [5, 7–9].

Ct causes infection in the lower genital tract. Untreated infected women may suffer from ascending infection, which can lead to complications such as chronic pain, inflammation and occlusion of the fallopian tubes, which may result in infertility and ectopic pregnancy [10, 11]. Ct increases the susceptibility and transmission of HIV-infection [12]. The infection is asymptomatic in up to 50% of women and 80% of men or may only display mild symptoms. Therefore, Ct infection often remains unnoticed and undiagnosed, and thus untreated [13].

Because of the considerable burden of Ct infection and its sequelae, particularly in young women, many countries have implemented Ct-related public health activities to enhance detection and to prevent negative consequences by treating infected individuals as soon as possible. Approximately half of the European countries offer opportunistic testing as a measure for Ct control. Only a few countries have a systematic approach with standard guidance for treatment and repeated testing [14]; however, there are guidelines on the management of Ct infection available for Europe [15]. Germany published a guideline in 2016 to enable optimal diagnostics and therapy of Ct [16]. In Germany, opportunistic screening has been offered free of charge for statutory insured pregnant women since 1995 and once annually for statutory insured sexually active women under 25 years of age since 2008. Currently, tests for men are refunded by statutory health insurance if carried out for diagnostic purposes, i.e. suspect infection based on symptoms or anamnesis. There is no screening program for men in Germany. There are no regulations for the contract tracing in Germany and usually it is performed. With the aim of gathering representative data on Ct tests, the Robert Koch-Institute (RKI) established a voluntary nationwide, laboratory-based Ct surveillance system, the ‘Ct laboratory sentinel’, in 2010 that accompanied the implementation of the Ct screening programme for women under 25 years old. Information on routine Ct-testing in combination with test results, reason for testing and patient-related information were collected from 24 microbiological laboratories in Germany offering Ct-diagnostics. The Ct laboratory sentinel covers 34% of all Ct tests performed in Germany. Study results based on the sentinel data found an overall positive result rate of 3.9% amongst women and 11.0% amongst men of all age groups [17]. Reinfection with Ct is possible and increases the likelihood of complications and future acquisition and transmission of the disease [18]. Based on international studies it was estimated that 13.9% of women and 11.3% of men have repeatedly been infected with Ct [19, 20] .

The association of previous infections, age and test reason on reinfection have not yet been analysed yet in Germany. The main aims of this study were to gather evidence on whether current infection was more frequent in women and men who had tested positive previously compared to those who had tested negative previously and, furthermore, to identify whether the age group and, for women, the reason for testing, along with previous test results, influences the result of a subsequent test. Our findings will contribute to identify special risk groups for Ct who are in need of health education and to generate evidence for targeted Ct testing programmes.

## Methods

The study population was composed of women and men who were tested for Ct in Germany between January 1, 2008 and December 31, 2014 and whose assays were tested in one of the laboratories participating in the Ct Laboratory Sentinel that provided traceable patient-IDs for a minimum of 3 years. The patient-IDs consisted of an encrypted 32-digit hash code. If patients were tested more than once within the same laboratory, data from several samples could be assigned by this unique identifier to one patient [16].

The following variables were used for analysis: patient-ID, assay-ID, laboratory code, testing date, age, sex, test result and test reason.

If information on sex or age was missing for some of the assays with the same patient-ID, the missing values were replaced by the information on sex and birth date available from the other assays. If sex was missing in all assays with the same patient-ID, these assays were excluded from analysis.

Multiple assays with the same patient-ID that were examined on the same day or within 7 consecutive days were considered as only one test. In this case, the test result was recorded as positive if at least one assay tested positive and recorded as negative if all assays were negative. The decision to summarize multiple assays was based on information regarding the time difference between the date when the sample was taken (available for 26% of all assays in the sample) and the date of testing. Assays taken the same day by the physician were tested within 7 days. Thus, multiple assays (such as vaginal and rectal swabs) belonging to the same test-event might have been tested in the laboratory on different days.

Assays with missing information on the test result were excluded from analysis. Assays with missing information on age group or reason for testing were kept in the analysis.

The nucleic acid amplification test (NAAT) that is predominantly used in Germany for Ct testing is very sensitive and can still be detected 3 weeks after therapy even though the pathogen is no longer vital [21, 22]. Thus, we included only those tests with a time interval of at least 30 days from the preceding test in the analysis. Reinfection was consequently defined as at least 2 positive laboratory tests within a time interval of more than 30 days, following the definition of Brunham et al. [23] and the European Guideline for the management of Ct which recommends a test of cure 4 weeks after completion of therapy [15].

We described the number and proportions of performed Ct tests, the number of tested persons and the number of tests per person by sex, age group, reason for testing and test result. Among repeat testers we calculated median time between Ct tests by initial and subsequent test result. “Initial test result” is defined as the first Ct test that we have captured in the sentinel, however, this might not be the first test of the individual.

We tested the association between the variables *previous test result* and *age group* and the outcome variable *test result* by using logistic regression. Because of the large sample size a *p*-value less than alpha = 0.01 was considered significant in all calculations. We included only tests from women and men at least 15 years old. Separate models were calculated for men and women, and for women, the analysis was additionally stratified according to the reason for testing. The persons tested only once in the surveillance period contribute to the calculation of the proportion tested positive. To include these persons into regression analysis, we defined the variable *previous test result* as “unknown” as this group is mixture of those previously never tested and those tested positive or negative. The stratified univariable analysis was compared to the results of a multivariable logistic regression model. We tested improved goodness of fit by using likelihood ratio tests (LRTs), and we calculated odds ratios (OR), probabilities (Pr), and the according 99% confidence intervals. Including the interaction in a model with two variables means that every combination of age group and previous test result was estimated separately. Since the result is a single odds it can directly be transformed into a probability (*P* = O/(O + 1)). We chose to present the probabilities, since for most people this is the more familiar measure. This implied that the probability of a positive test result had to be estimated separately in all strata. The data were extracted from an SQL dataset and analysed using STATA 14.

## Results

### Study population

#### General characteristics of Ct testing in Germany

**Fig. 1:**
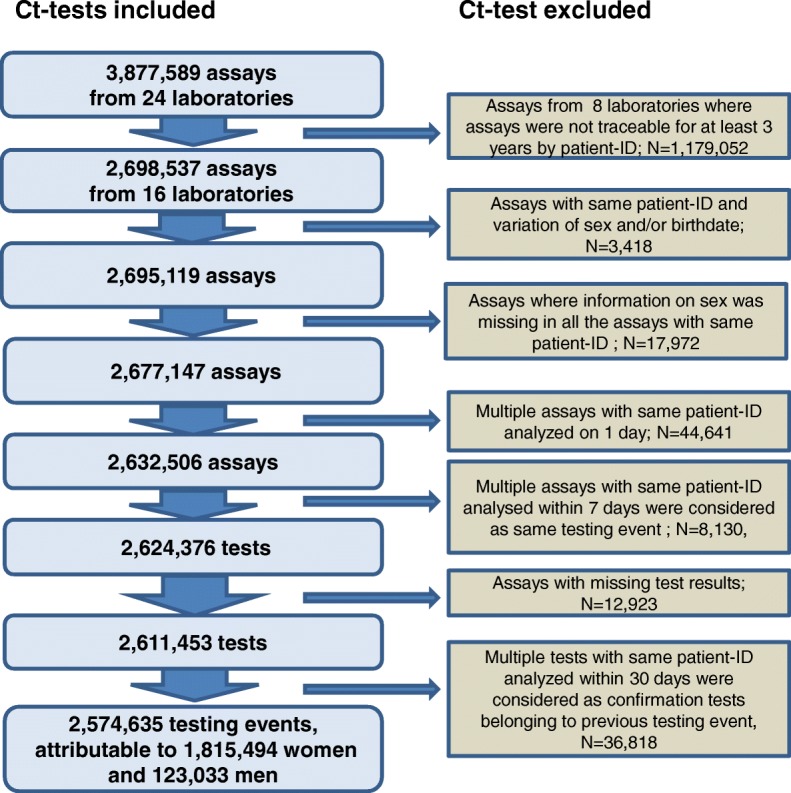
Flowchart of sample selection

During 2008–2014, 3,877,589 Ct tests were reported. Of those, 2,574,635 tests were analysed, including 2,429,942 in women and 144,693 in men (Fig. 1). These tests could be attributed to 1,815,494 women and 123,033 men. The median age at the first Ct test was 26 years in women (IQR: 22–32) and 33 years (IQR: 25–43) in men. Note that this might not be the first test of the individual. In women, most tests (45.0%) were performed because of screening in pregnancy, followed by screening under 25 years (27.9%) and diagnostic testing (27.2%). The median age at the time of the first positive test was 22 years for women (IQR: 19–25) and 28 years for men (IQR: 23–36). Number of Ct tests and proportion of positive Ct tests by reason of testing and age group among women and men is given in Table 1.

**Table 1:** Number of Ct tests and proportion of positive Ct tests by reason of testing and age group among women and men, 2008–2014, *n* = 2,574,635

	Women		Men	
	Number (Proportion in %)	Proportion Ct positive in %	Number (Proportion in %)	Proportion Ct positive in %
Tests				
Total	2,429,942 (100)	4.0	144,693 (100)	12.4
Reason for testing (tests)				
Screening under 25 years	571,054 (23.5)	5.0	n. a.	n. a.
Screening in pregnancy	920,133 (37.9)	2.5	n. a.	n. a.
Diagnostic testing	555,853 (22.9)	4.6	144,693 (100)	12.4
Unknown	382,902 (15.8)	5.1	n. a.	n. a.
Age group (tests)				
0–15 years	13,106 (0.5)	3.4	1048 (0.7)	3.0
15–19 years	342,892 (14.1)	6.8	6634 (4.6)	16.6
20–24 years	736,040 (30.3)	6.0	24,545 (17.0)	21.2
25–29 years	494,432 (20.4)	3.3	24,924 (17.2)	17.1
30–34 years	445,559 (18.3)	1.6	21,165 (14.6)	12.1
35–39 years	238,306 (9.8)	1.2	17,211 (11.9)	9.3
40+ years	157,824 (6.5)	1.3	46,868 (32.6)	5.7
Unknown	1783 (0.1)	5.1	2298 (1.6)	7.7

Altogether, 23.1% (*n* = 420,220) of tested women and 11.9% (*n* = 14,680) of tested men were tested more than once during the 7-year-surveillance period. Most of those were tested twice (69.5% of women and 73.6% of men). While 20.1 and 10.4% of women and 16.4 and 10.0% of men were tested 3 and 4 or more times, respectively.

Amongst women and men tested more than once 13.6% (*n* = 57,133) and 34.6% (*n* = 5073) respectively had at least one positive Ct test. In women and men, the median time until the date of the subsequent test was shorter if the previous test was positive (Table 2).

**Table 2:** Median time interval until subsequent test in women and men stratified by previous test result, 2008–2014, *n* = 1,074,430

Time interval until subsequent test	Women	Men
	Median time in months (IQR)	Median time in months (IQR)
Negative test	15.5 (IQR: 9.4–25.6)	7.4 (IQR: 3.1–15.2)
Positive test	3.0 (IQR: 1.7–8.9)	2.8 (IQR: 1.5–8.1).

Overall, in 0.5% (*n* = 8369) of women and in 0.8% (*n* = 963) of men in all the tested men and women reinfection was detected. Amongst repeatedly tested women and men, reinfection was detected in 2.0 and 6.6%, respectively. The median time from the first to the second positive test (reinfection) was 4.2 months in women (IQR: 2.1–13.0) and 6.7 months in men (IQR: 2.3–16.6).

#### Uni- and multivariable analysis

**Fig. 2:**
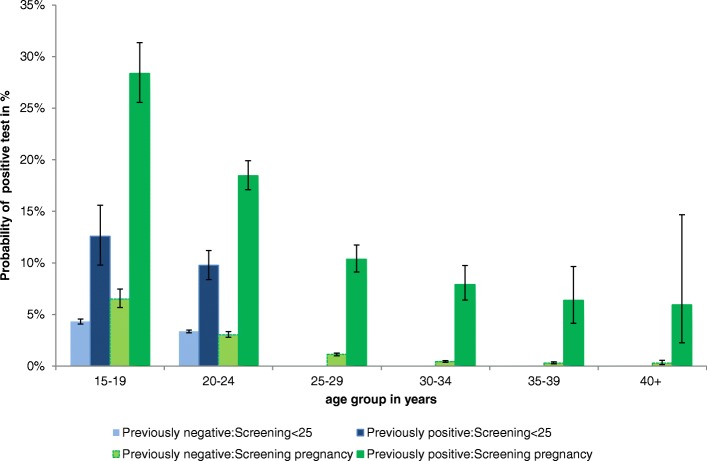
Probability and 99%-CI of positive test results according to age group and previous test results in women tested in the frame of screening programmes by age group, 2008–2014

**Fig. 3:**
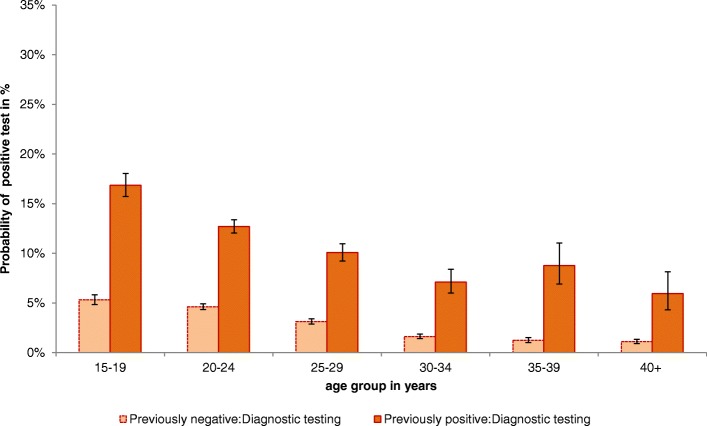
Probability and 99% CI of positive test result according to age group and previous test results in women tested for diagnostic reasons, 2008–2014

**Fig. 4:**
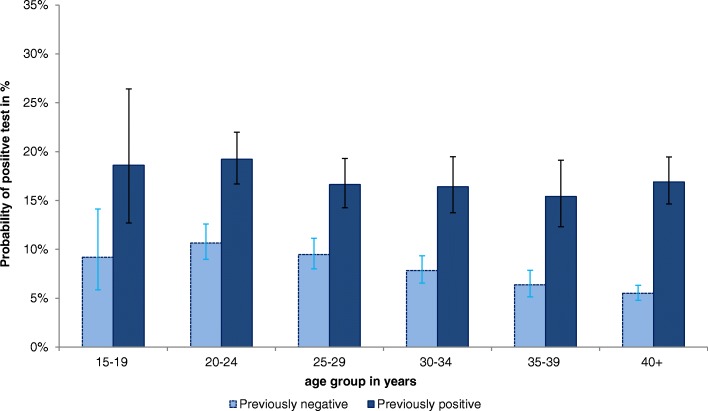
Probability and 99% CI of positive test result according to age group and previous test results in men, 2008–2014

In the univariable logistic regression, the variables *age group* and *previous test result* were significantly associated with a positive result in a subsequent test (for detailed results of univariable analysis for women see Additional file 1: Table S1). A multivariable logistic regression model including the variables *age group* and *previous test result* showed that their interaction had a significant effect (Likelihood-Ratio-Test: *p* < 0.01). In regard to the probability of a positive test result, a similar result was reported in women for each test reason. Women and men of all age groups who had previously tested positive showed a higher probability of testing positive compared to their peers who had previously tested negative. The estimated probabilities for women and men are shown in Table 3 and Figs. 2, 3 and 4. A different presentation of the results for women and men in odds ratios can be found in Additional file 2: Figure S1 and Additional file 3: Figure S2.

**Table 3:** Probability (Pr) of positive test result by previous test result and age group among women according to reason for testing and men, 2008–2014

Previous test result	Age group, years	Pr to test positive in % (99%-CI)			
		Women			Men
		Screening under 25 years	Screening in pregnancy	Diagnostic testing	All tests
Negative	15–19	4.3 (4.1–4.6)	6.5 (6.0–8.1)	5.3 (4.8–5.8)	9.2 (5.9–14.1)
	20–24	3.4 (3.2–3.5)	3.0 (2.9–3.4)	4.6 (4.3–4.9)	10.7 (9.0–12.6)
	25–29	–	1.1 (1.0–1.3)	3.1 (2.9–3.4)	9.5 (8.0–11.2)
	30–34	–	0.4 (0.4–0.5)	1.6 (1.4–1.9)	7.8 (6.5–9.3)
	35–39	–	0.3 (0.2–0.4)	1.2 (1.0–1.5)	6.4 (5.2–7.9)
	40+	–	0.3 (0.1–0.6)	1.1 (0.9–1.3)	5.5 (4.8–6.3)
Positive	15–19	12.6 (11.3–14.0)	28.4 (34.3–45.7)	16.8 (15.7–18.1)	18.6 (12.7–26.4)
	20–24	9.8 (9.1–10.4)	18.5 (20.6–24.9)	12.7 (12.0–13.4)	19.2 (16.7–22.0)
	25–29	–	10.4 (10.0–13.3	10.1 (9.2–11.0)	16.6 (14.3–19.3)
	30–34	–	7.9 (6.8–10.8)	7.1 (6.0–8.4)	16.4 (13.8–19.5)
	35–39	–	6.4 (4.3–10.7)	8.8 (6.9–11.0)	15.4 (12.3–19.1)
	40+	–	5.9 (2.3–17.2)	5.9 (4.3–8.1)	16.9 (14.7–19.4)
Unknown	15–19	4.9 (4.7–5.0)	9.7 (10.2–11.3)	8.1 (7.7–8.4)	16.9 (15.7–18.2)
	20–24	5.7 (5.6–5.8)	5.8 (6.0–6.4)	8.3 (8.0–8.6)	22.3 (21.6–23.1)
	25–29	–	2.2 (2.1–2.3)	5.1 (5.0–5.3)	18.0 (17.3–18.7)
	30–34	–	1.1 (1.0–1.1)	2.9 (2.7–3.0)	12.4 (11.8–13.1)
	35–39	–	0.7 (0.6–0.8)	1.9 (1.8–2.1)	9.4 (8.8–10.1)
	40+	–	0.6 (0.5–0.8)	1.6 (1.5–1.7)	6.1 (5.8–6.4)

In women who were tested in the frame of screening programmes (Table 3, Fig. 2), the probability to test positive generally decreased with increasing age. The proportion of previously positive pregnant women testing positive again was highest amongst the 15–19 and 20–24-year-olds. However, the difference in regard to the previous test result was most pronounced in pregnant women in older age groups. Previously positive tested women aged 35–39 years were 21.1 times and women aged 40+ 20.5 times more likely to test positive compared to their previously negative tested peers.

Amongst diagnostic tests the probability to test positive was highest in the age groups 15–19 and 20–24 years in women who had previously tested positive. Overall, the probability to test positive decreased with increasing age, regardless of the previous test result. We observed a slight increase in the probability of a positive test result in the group of previously positive 35- to 39-year-olds. The difference between women who had previously tested positive and their previously negative tested peers was most pronounced in the age groups 35–39 and 40+ years (OR: 7.1 and 5.4, respectively).

The probability to test positive in men (Table 3, Fig. 4) was highest amongst 15–19 and 20- to 24-year-olds who had previously tested positive. The probability to test positive decreased with increasing age; however, amongst men who had previously tested positive, we observed a slight increase in the age group 40+. The difference between previously positive and previously negative tested men was most distinct in the age groups 35–39 and 40+ years (OR: 2.6 and 2.8, respectively).

## Discussion

To our best knowledge, this was the first German study using a comparably large dataset of routine Ct tests to examine the likelihood of infection while taking into account the result of the previous test.

The likelihood of being diagnosed with Ct differed based on the reason for testing and age in women. In particular, young pregnant women who had a history of Ct infection were at a high risk to test positive. As in Germany the mean age of women at the time of the birth of their first child was 29.3 years in 2015 [26], it is possible that many pregnancies in the age group 15–19 years were unwanted and screening for Ct has been carried out in the context of an abortion and can be related to behaviour with higher risk to acquire a STI as discussed before [17].

The proportion of positive tests was considerably higher in men compared to women.

This can be explained by the fact that men are usually tested because of symptoms or anamnestic reasons. Furthermore, past analysis of the Ct laboratory sentinel showed that a substantial proportion of tested material is rectal swabs [27] . Based on previous national and international studies, it is known that the proportion of positive tests is higher among MSM than heterosexual men [13, 29]. This aspect might have influenced the data we observe.

Less than one fourth of all tested women and men were tested repeatedly during the study period. Because screening for women < 25 years of age should be performed annually, and women can also be tested because of screening in pregnancy and for diagnostic reasons, we expected higher proportion of repeatedly tested women. The small proportion of repeatedly tested women suggests that screening programmes might not be sufficiently implemented in Germany yet. To tackle insufficient coverage of the existing screening programs, the Federal Agency for Health Education (BZgA) designed an information campaign and information materials on chlamydia screening for physicians and attendees of medical praxes, in addition to an ongoing poster campaign on STIs and corresponding symptoms [28].

In all age groups and regardless of the reason for testing, women and men with prior Ct infection had a high probability to be tested positive for Ct (women: Pr: 5.9–28.4%, men: Pr: 15.4–19.2%). The probability of reinfection varied substantially among women by age and test reason with highest probabilities in young women, especially if tested within screening in pregnancy. Studies have shown that early first intercourse was associated with subsequent sexual risk behavior [9, 24] which also leads to a higher probability of acquiring an STI. We observed less age-related variation for reinfection among men, though younger men had a higher probability for reinfection. We believe that this might be related to a mixed population of men in our data set. The group that repeatedly tested positive might be more at risk of infection, for example HIV positive MSM. Bacterial STI are more common in HIV positive MSM [13, 30]. However, we lack data in current study to analyse this in more detail. Additionally, the detection rate of STI, especially asymptomatic infections, among men frequently accessing testing might be higher.

Currently, evidence remains insufficient to recommend routine Ct-screening for all sexually active young men in Germany, partially because men rarely develop sequelae [31]. Nonetheless, the benefit of a screening programme for high-risk populations of men has been supported, for example Gift et al. found that Ct-screening programme targeting high-risk men was cost-saving compared to programme expanding screening of lower-risk women [32].

Interestingly, previously positive tested women and men in the older age groups showed the highest odds ratios to test positive; women especially when screened in pregnancy. This finding could indicate that these women and men aged 30 years and older with repeat infection, may represent a small group with ongoing sexual risk behavior that is thus more likely to contract repeat infection than their previously negative tested peers. Nevertheless, the absolute risk differences (in percent points) between previously positive and previously negative tested women and men by age group are much higher in the young age groups. The increased probability of infection for women and men with a history of Ct infection has been found by several other studies. In a study population of adolescent girls, Batteiger et al. found that incident Ct infections occurred in 78.1% of participants with infections at baseline compared to 51.7% of participants without infection at baseline [33]. The results of Dunne et al. showed a 1.2-fold risk for men with previous Ct infection amongst men screened for Ct infection [34], whereas Rietmejer et al. found a 2.4-fold increased risk of reinfection for women and men with Ct infection at baseline amongst female and male patients of an STI clinic [25]. However, because of wide variations amongst the definitions of reinfection, the study designs and the composition of study populations, it is difficult to compare these numbers directly to our results.

Untreated infections of current partners and unprotected sex with new partners contribute to repeated infections [17, 35]. Although treatment with antibiotics is highly effective [33], a small proportion of alleged reinfection might be ongoing infections after treatment failure or insufficient adherence to treatment. Several studies have shown that persons with prior Ct infection were at a high risk of reinfection [9, 20, 25, 33, 34]. However, a link between previously diagnosed and treated Ct infections and the development of immunity against Ct has also been discussed [23].

The testing interval was considerably shorter if a previous Ct test was positive. A median time to repeat testing after a positive test result of approximately 3 months might indicate compliance to European guideline that recommends repeat testing in women and men < 25 years of age within 3 to 6 months after a positive Ct test result [15]. Additionally, test of cure is recommended for pregnant women, if symptoms persist, if non-compliance is suspected or if second- and third-line treatments have been used [15]. However, the median time to the reinfection depends largely on the testing patterns and cannot directly be related to the natural history of infection.

The relatively small proportion of persons with reinfection we found in our whole sample was likely a consequence of the small proportion of repeatedly tested persons in our sample. While among those tested repeatedly during the study period, the reinfection was observed in 2% of women and 6.5% of men. The median time to reinfection was 4.2 month in women and 6.7 months in men. Similar to our results, studies from the United States and Australia have found a median time to reinfection of 5.2 months amongst 19-year-old women [29] and of 4.6 months amongst women aged 16 to 25 years [9]. The treatment failure can still be observed 7–8 weeks after antibiotic treatment. Therefore, it is important to be able to distinguishing between actual reinfection with Ct and treatment failure [36]. As we have no information on treatment and detailed follow up data, we cannot distinguish between treatment failure and reinfection. Therefore, our approach to define a reinfection as each infection that occurs after a 30 days period is sensitive and potentially misclassifies those with treatment failure. In a sensitivity analysis, where we defined a reinfection as a subsequent positive test occurring after 42 days, we observed a reinfection in 1.9% (instead of 2.0%) of repeatedly tested women and in 6.4% (instead of 6.6%) of repeatedly tested men. With this approach, it is probable that some of the true reinfections are misclassified treatment failures.

### Limitations

First, because several samples of a person only received the same patient-ID if tested in the same laboratory, the true number and proportion of reinfection in the study population may have been underestimated. If samples of one person were sent to different laboratories, e.g., because someone changed physician or the physician changed the diagnosing laboratory, these persons were either lost to follow-up or their tests were sampled in the Ct laboratory sentinel again, but with a new patient-ID. However, we expect that the proportion of previously tested persons having received a new patient-ID represents only a small fraction of the analysed tests.

Another limitation is that the *Ct laboratory sentinel* dataset offers no information on whether a person who tested positive received therapy and whether the treatment was successful. Thus, ongoing Ct-infections due to non-compliance or treatment failure could have been falsely classified as reinfection in this study. Furthermore, there is no information on behavioural factors that may influence the likelihood to be tested positive.

Finally, the Ct Laboratory Sentinel data might lack representativeness for the target population in Germany, even though the sentinel generally reached a good coverage in the period 2008–2014 and it was possible to collect a large number of samples representing one third of all performed Ct tests in Germany [17]. Still, the participation of laboratories was voluntary and it is unclear whether participating laboratories differed systematically from the non-participating laboratories, e. g. in terms of tested population. Data from the laboratories with continuous follow-up for at least 3 years used in current analysis did not substantially differ from rest of the participating laboratories regarding distribution of sex, age and proportion tested positive.

## Conclusions

We describe how history of Ct infection increased the likelihood of infection with Ct in women and men, taking into account age and reason for testing (in women). This helps to identify groups in need of health education and generates evidence for a more targeted testing strategy.

The high proportion of positive tests and of reinfection found amongst men supports risk-based screening of sexually active young men. However, future studies must determine all potential risk factors and identify the categories of transmission in men to identify for which group Ct screening is most beneficial.

We recommend that women and men who have been diagnosed positive with Ct should be consulted on prevention, symptoms and possible consequences of Ct infection to avoid reinfection.

To identify potential reinfection – and persisting infection – repeat testing after treatment should be performed. To increase the number of those getting tested, preventive measures for young women and men like awareness campaigns on Ct infection and testing opportunities as well as sex education in schools should be implemented. Till now, preventional campaigns for Ct in Germany do not address a higher risk for infection of persons who were tested positive before specifically. On basis of our data, we recommend to adjust the respective materials to emphasize this. Awareness about Ct infection, increased risk of reinfection for person tested Ct positive before, and adaequate screening should also be raised amongst physicians and gynaecologists to enhance the coverage of the screening programmes. Further studies are necessary to examine whether these measures show the desired effect.

## Additional files

Additional file 1: **Table S1.** Univariable association of previous test result and age-group with “tested Ct positive” by test reason among women, 2008–2014. (DOCX 15 kb)

Additional file 2: **Figure S1.** Odds Ratio and 99%-CI: Positive test results in previously positive vs. previously negative tested women by age group and test reason. Source: Ct Laboratory Sentinel 2008–2014. (DOCX 47 kb)

Additional file 3: **Figure S2.** Odds Ratio and 99%-CI: Positive test results in previously positive vs. previously negative tested men by age group and test reason. Source: Ct Laboratory Sentinel 2008–2014. (DOCX 37 kb)

## Abbreviations

CDC: Centers for Disease Control and Prevention
Ct: Chlamydia trachomatis
ECDC: European Center for Disease Control and Prevention
LRT: Likelihood ratio test
MSM: Men having sex with men
NAAT: Nucleic acid amplification test
OR: Odds ratio
Pr: Probability
STI: Sexually transmitted infection
