# Supplementary material for: Not again! Effect of previous test results, age group and reason for testing on (re-)infection with Chlamydia trachomatis in Germany
Source: BMC Infect Dis. 2018 Aug 25;18:424. doi: 10.1186/s12879-018-3323-2 (PMC6109262; doi:10.1186/s12879-018-3323-2)
Supplement: Supplementary file 1 — Table S1. Univariable association of previous test result and age-group with “tested Ct positive” by test reason among women, 2008–2014. (DOCX 15 kb) [file 12879_2018_3323_MOESM1_ESM.docx]

**Table S1**: Univariable association of previous test result and age-group with “tested Ct positive” by test reason among women, 2008-2014

|  |  | Odds ratio | p-value | lower 99%-CI | upper 99%-CI |
| --- | --- | --- | --- | --- | --- |
| Test reason: Screening <25 | | | | | |
| Previous test results | negative | Ref |  |  |  |
|  | positive | 3.08 | 0.000 | 2.87 | 3.31 |
|  | unknown | 1.49 | 0.000 | 1.43 | 1.55 |
| Age group | 20-24 years | Ref |  |  |  |
|  | 15-19 years | 0.96 | 0.004 | 0.93 | 0.99 |
|  | | | | | |
| Test reason: Screening pregnancy | | | | | |
| Previous test results | negative | Ref |  |  |  |
|  | positive | 13.95 | 0.000 | 12.79 | 15.22 |
|  | unknown | 1.94 | 0.000 | 1.82 | 2.06 |
| Age group | 20-24 years | Ref |  |  |  |
|  | 15-19 | 1.82 | 0.000 | 1.73 | 1.92 |
|  | 25-29 | 0.35 | 0.000 | 0.34 | 0.37 |
|  | 30-34 | 0.16 | 0.000 | 0.16 | 0.17 |
|  | 35-39 | 0.10 | 0.000 | 0.10 | 0.12 |
|  | 40 years and older | 0.10 | 0.000 | 0.08 | 0.12 |
| Test reason: Diagnostic testing | | | | | |
| Previous test results | negative | Ref |  |  |  |
|  | positive | 4.34 | 0.000 | 4.09 | 4.61 |
|  | unknown | 1.45 | 0.000 | 1.38 | 1.52 |
| Age group | 20-24 years | Ref |  |  |  |
|  | 15-19 | 1.08 | 0.000 | 1.03 | 1.13 |
|  | 25-29 | 0.62 | 0.000 | 0.59 | 0.65 |
|  | 30-34 | 0.33 | 0.000 | 0.31 | 0.35 |
|  | 35-39 | 0.23 | 0.000 | 0.21 | 0.25 |
|  | 40 years and older | 0.19 | 0.000 | 0.17 | 0.20 |
