# Supplementary figures and images for: Not again! Effect of previous test results, age group and reason for testing on (re-)infection with Chlamydia trachomatis in Germany
Source: BMC Infect Dis. 2018 Aug 25;18:424. doi: 10.1186/s12879-018-3323-2 (PMC6109262; doi:10.1186/s12879-018-3323-2)

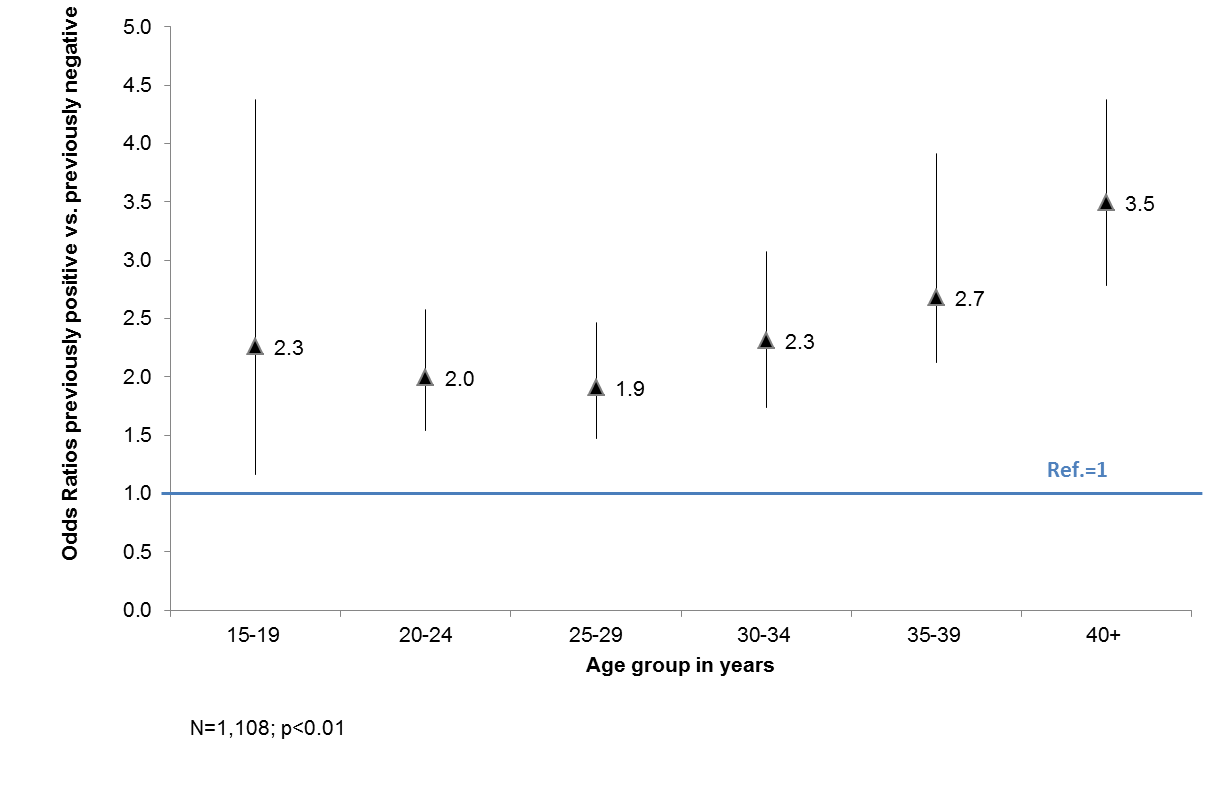

Supplement: Supplementary file 2 — Figure S1. Odds Ratio and 99%-CI: Positive test results in previously positive vs. previously negative tested women by age group and test reason. Source: Ct Laboratory Sentinel 2008–2014. (DOCX 47 kb) [file 12879_2018_3323_MOESM2_ESM.docx]

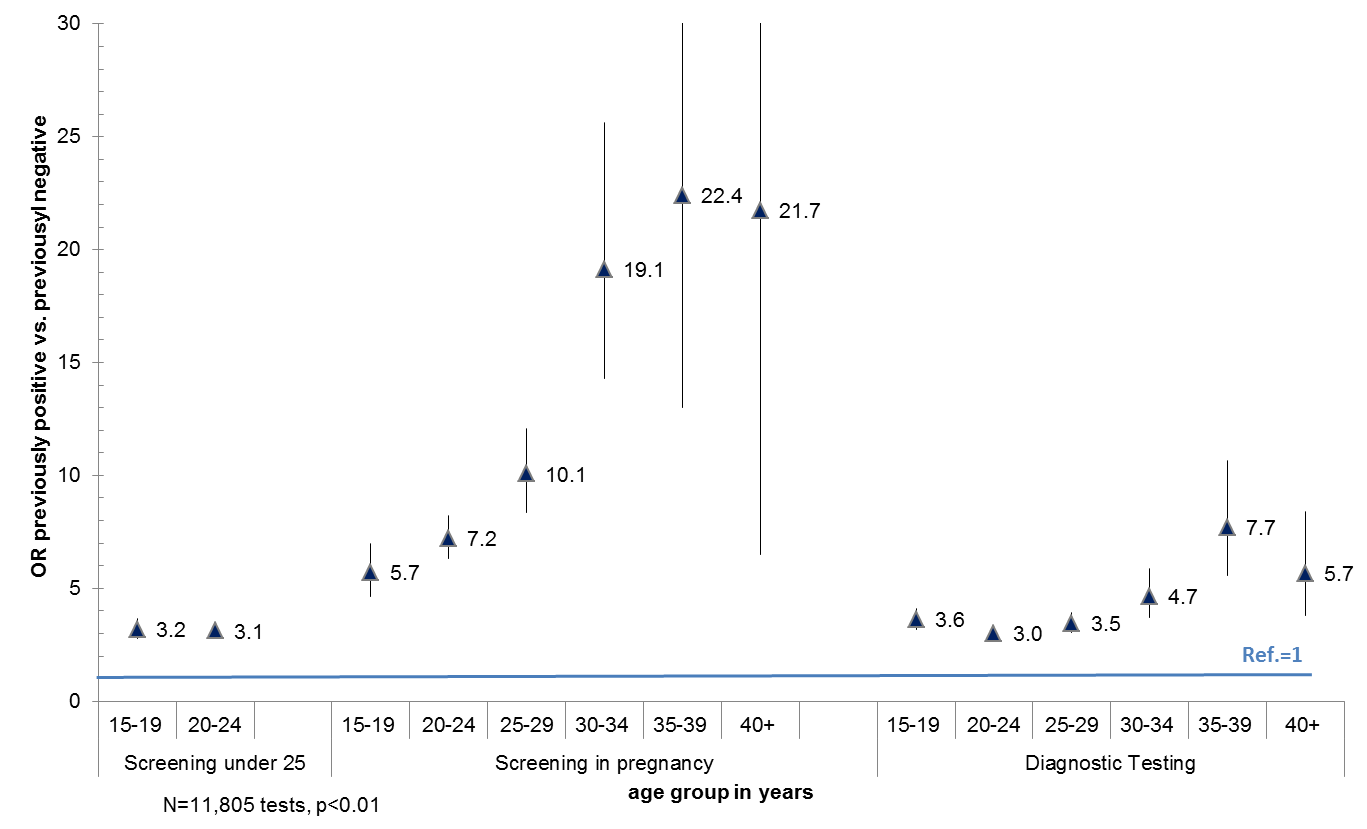

Supplement: Supplementary file 3 — Figure S2. Odds Ratio and 99%-CI: Positive test results in previously positive vs. previously negative tested men by age group and test reason. Source: Ct Laboratory Sentinel 2008–2014. (DOCX 37 kb) [file 12879_2018_3323_MOESM3_ESM.docx]
